# Diverse MHC IIB allele repertoire increases parasite resistance and body condition in the Long-tailed giant rat (*Leopoldamys sabanus*)

**DOI:** 10.1186/1471-2148-9-269

**Published:** 2009-11-23

**Authors:** Tobias L Lenz, Konstans Wells, Martin Pfeiffer, Simone Sommer

**Affiliations:** 1 Department of Animal Ecology and Animal Conservation, University of Hamburg, 20146 Hamburg, Germany; 2 Department of Evolutionary Ecology, Max Planck Institute for Evolutionary Biology, 24306 Plön, Germany; 3 Institute of Experimental Ecology, University of Ulm, 89069 Ulm, Germany; 4 Evolutionary Genetics, Leibniz-Institute for Zoo and Wildlife Research (IZW), Postfach 601103, D-10252 Berlin, Germany

## Abstract

**Background:**

Genes of the major histocompatibility complex (MHC) code for key functions in the adaptive immune response of vertebrates and most of them show exceptionally high polymorphism. This polymorphism has been associated with the selection by diverse and changing parasite communities. We analysed MHC class IIB diversity, gastrointestinal parasite load and body condition in the wild ranging tropical rat *Leopoldamys sabanus *(Thomas, 1887) under natural selection conditions in a highly variable rainforest environment in Borneo to explore the mechanisms that maintain these high levels of genetic polymorphism.

**Results:**

Allelic diversity was determined via SSCP and sequencing, and parasite screening was done through non-invasive faecal egg count. The detected alleles showed expected high levels of polymorphism and balancing selection. Besides a clear advantage for more diverse MHC genotypes in terms of number of alleles, reflected in better body condition and resistance against helminth infection, our data also suggested a positive effect of MHC allele divergence within an individual on these parameters.

**Conclusion:**

In accordance with the heterozygote advantage hypothesis, this study provides evidence for an advantage of more diverse MHC genotypes. More specifically, the potential negative relation between individual allele divergence and number of parasite species is in line with the '*divergent allele advantage*' hypothesis.

## Background

Genetic variation is the material for evolutionary processes. Its maintenance may to a large part be driven by the exceptional dynamics of host-parasite co-evolution [1,2]. The major histocompatibility complex (MHC) represents a crucial part of the host side in these co-evolutionary processes and is one of the most polymorphic systems in the vertebrate genome. It has been studied extensively in model species under laboratory conditions [3], but also became the focus of an increasing number of studies on natural populations because of its functional importance for the immune system and mate choice [4-6]. Its genes code for cell surface molecules that present self and non-self antigens to T-cells [7]. This function makes them a key factor of the adaptive immune system as it triggers a respective targeted response upon recognition of non-self antigens and therefore plays a vital role in the recognition of pathogens invading the body. The high polymorphism in this system, which is composed of a high total number of alleles as well as allelic divergence on the sequence level, is thought to facilitate populations to persist in and adapt to natural habitats [5,8].

A number of hypotheses have been proposed to explain the forces that counteract the effects of random genetic drift and fixation [reviewed in [5,6,9-11]]. The '*negative frequency-dependent selection*' hypothesis [12-14] grounds on the effects of host parasite co-evolution ['Red Queen hypothesis', [15]], stating that frequencies of alleles constantly change with the frequency of adapted and non-adapted pathogens. Rare alleles are being preserved within a population through such fluctuations or the emergence of new pathogens. The prerequisite for this co-evolution in terms of reciprocal adaptation is the direct interaction of individual alleles and distinct pathogenic morphotypes. Growing evidence comes from a variety of taxa that such an interaction exists [16-23]. Another hypothesis, the *'heterozygote advantage*' has been proposed as a general mechanism to maintain high allele numbers in populations by an advantage for individuals carrying more than one allele, enabling them to present a broader range of antigens [24,25]. There is experimental evidence that this is valid for animals exposed to multiple pathogens [[26], up to an optimum in [27]] but not to single pathogens [28]. This seems plausible, as the advantage would most likely result from a combined resistance of individual alleles each presenting a certain range of parasites [29,30]. Nevertheless, the exact mechanisms are still a matter of debate [31,32]. De Boer *et al*. [33] used mathematical models to study the degree of MHC polymorphism arising when '*heterozygote advantage*' is the only selection pressure. The simulations revealed that the advantage for an individual that simply carries two alleles instead of one is not sufficient to explain the high population diversity of the MHC. Wakeland *et al*. [34] proposed a special case of the heterozygote advantage, the '*divergent allele advantage*', which refers to the genetic distance between alleles and expects an advantage for more divergent allele combinations, enabling the respective carrier to present more different antigens to its adaptive immune system. This mechanism may potentially explain a directional selection for both, a large number of alleles at the population level and a high divergence of alleles at the individual level.

Tropical rainforests are known for their exceptional species diversity [35] and this may also be true for the diversity of parasites an inhabitant has to face. Therefore studying a species from such diverse environment could provide a great opportunity to better understand the ongoing evolution of the MHC, as selection mechanisms should be pronounced. In this study we examine the variability in the exon 2 of MHC class IIB genes and its effect on parasite infection of the Long-tailed giant rat (*Leopoldamys sabanus*) to unravel the mechanisms of selection in a wild ranging species. This large nocturnal murid species lives semi-arboreal in tropical forested habitats and reaches an average body mass of 368 g [36], feeding on plant material and arthropods [37]. This rodent was chosen, because it is a wide-spread species living in the diverse and dynamic environment of dipterocarp rainforests across Southeast Asia [38,39], presumably conferring the need to constantly adapt to environmental changes such as the encounter of new parasites, but also to persist against ancient pathogen lines. The presumably high parasite pressure in these habitats could lead to pronounced and therefore detectable signs of elsewhere more subtle mechanisms of selection.

## Results

### MHC II gene variability

In total we determined the MHC class IIB genotype of 46 individuals from the two different subpopulations. Overall 28 different sequence variants (alleles) could be distinguished via SSCP and confirmed by sequencing. The genomic origin of the alleles was investigated through a protein BLAST search on the NCBI database [40]. Ten of the detected alleles showed unique DRB origin, blasting to different murid species, ten other alleles produced hits with DRB as well as DQB alleles from different murid species and the last eight alleles produced mainly DQB hits from several murid taxa. The same latter eight alleles showed a deletion of two amino acids, leading to a shortened amino acid sequence, but not to a frame shift in codon translation. One of the two deleted amino acid sites (position 65) is thought to be involved in antigen binding [41]. The same deletion can also be found in some (but not all) published DQB alleles of the Brown rat (*Rattus norvegicus*; e.g. [GenBank:AY626188]) and House mouse (*Mus musculus*; e.g. [GenBank:AY740472]).

**Figure 1:**
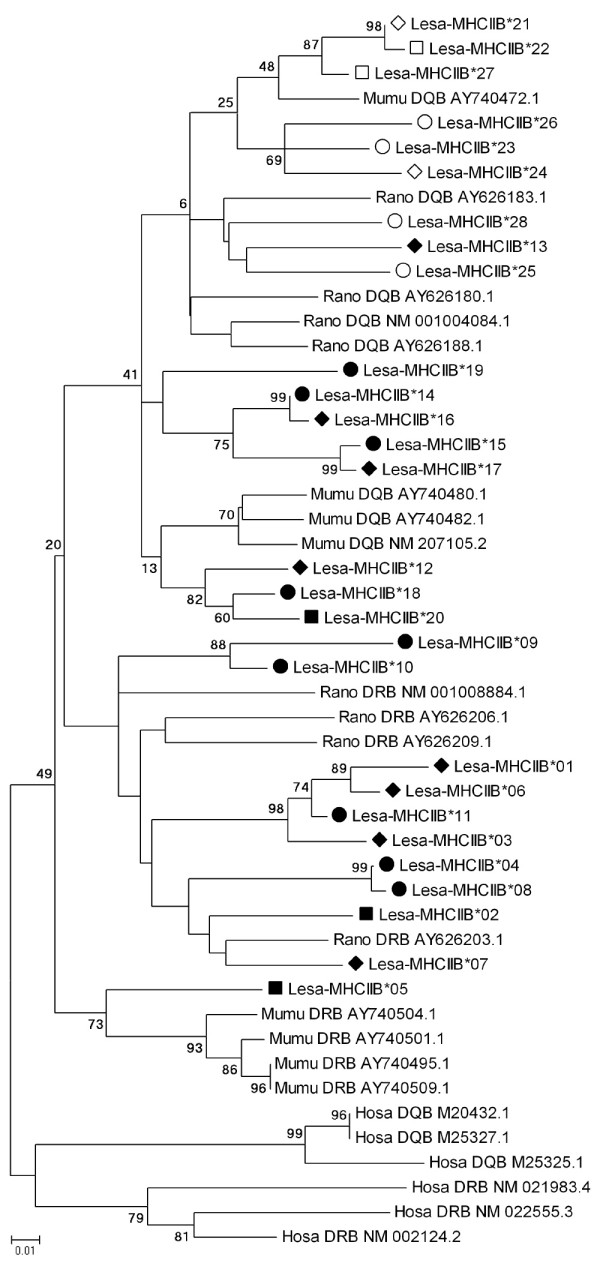
**Neighbour-joining tree (bootstrap consensus) of MHC class IIB alleles**. Phylogenetic tree based on NJ algorithms calculated at the nucleotide level; including *Leopoldamys sabanus *(Lesa) and non-Lesa alleles (Hosa - *Homo sapiens*, Rano - *Rattus norvegicus*, Mumu - *Mus musculus*). Alleles occurred in both subpopulations (◆), only in Poring (■) or only in Monggis (●). Open symbols indicate alleles with a deletion of two amino acids. For the non-basal nodes, only bootstrap values above 50 are shown.

Due to the ambiguous information from other species, we did not assign locus origin to any of the alleles. Instead we labelled them in the order of discovery with the prefix Lesa-MHCIIB and submitted them to GenBank [GenBank:EU543225 - EU543252]. Although several of the alleles show ambiguities concerning their origin, we did not find traces of gene conversion events between the potential two loci using GENECONV (no inner or outer fragments detected, global SimP-values p = 0.155 and p = 0.387 respectively). We produced a neighbour-joining tree of all alleles, including published DRB and DQB alleles from rat, mouse and human (Maximum Composite Likelihood model for nucleotides, 10000 iterations, starting seed: 90572, excluding deletions and allowing for heterogeneous patterns among lineages). In contrast to the included human DRB and DQB alleles, which showed strong locus divergence, the murid alleles did not cluster in a significant pattern (Fig. 1), suggesting that the DRB and DQB lineages in murids are not as differentiated as they are in primates. The same holds true when including the deleted sites in a pair wise comparison (tree not shown). Due to these results and because both potentially amplified loci code for classical MHC class II molecules which present extracellularly derived antigens, we combined all detected alleles for analysing possible relationships of MHCIIB diversity with parasite loads and body condition.

**Figure 2:**
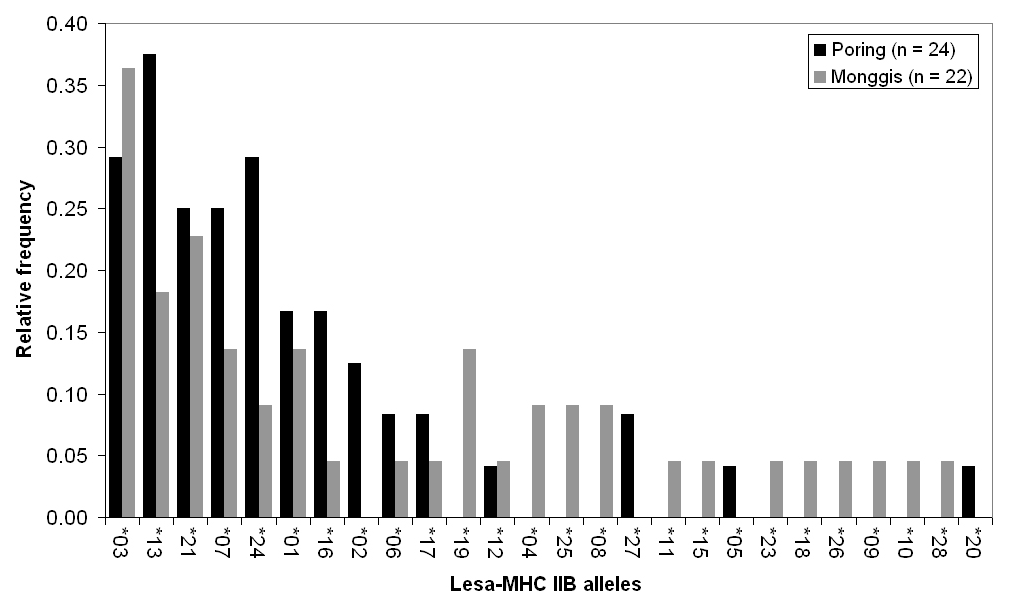
**Frequencies of Lesa-MHC IIB alleles in both study sites (Poring and Monggis)**. The frequency is expressed in relative number of individuals per study site in which the respective allele occurred. The order of the alleles reflects overall frequency. The allele prefix 'Lesa-MHCIIB' has been omitted for simplification. The two alleles Lesa-MHCIIB*14 and Lesa-MHCIIB*22 that showed only synonymous substitutions occurred in one and three individuals, respectively, and were merged with their synonymous counterparts Lesa-MHCIIB*16 and Lesa-MHCIIB*21.

The frequency of alleles differed strongly (Fig. 2). While most (58%) of the alleles were rare and occurred only in one or two individuals, the most common allele Lesa-MHCIIB*03 was found in one third of all screened animals. The two alleles Lesa-MHCIIB*14 and Lesa-MHCIIB*22 equalled the alleles Lesa-MHCIIB*16 and Lesa-MHCIIB*21 respectively after translation into amino acid sequence, due to only synonymous substitutions, leaving 26 different alleles on the amino acid level. Synonymous alleles were pooled for subsequent analyses, however, none of them occurred together in the same individual.

The two populations shared 82% of those alleles, which were present in more than two individuals (9 out of 11) and none of the private alleles, i.e. unique to one of the study sites, occurred in more than 3 individuals (Fig. 2). Genotypes ranged from one to four alleles per individual. We calculated the divergence of alleles for each individual, which ranged from 0 to 0.404 and correlated strongly with the number of alleles (nonparametric correlation, Spearman's rho = 0.58, p < 0.001).

### Evidence for selection

Within the alleles, 70% of the amino acid positions appeared variable and non-synonymous substitutions occurred in a significant higher magnitude than synonymous ones (d_N_/d_S _= 1.78, Z-test, p = 0.018; Table 1), especially in the regions that code for the antigen binding sites (ABS; d_N_/d_S _= 7.6, Z-test, p < 0.001). The same increased d_N_/d_S _ratio at the ABS was also found in a separate analysis of only the eight alleles with a deletion (Z-test, p = 0.002; Table 1). Such elevated d_N_/d_S_-ratios are a widely accepted sign for balancing selection in polymorphic genes [42,43]. These results were confirmed by the maximum likelihood approach in CODEML, showing a significantly higher likelihood estimate for the model M8 which allows for positive selection compared to the model M7 which assumes no positive selection (2Δl = 76.69, df = 2, p < 0.0001). The site class under positive selection showed an ω (d_N_/d_S _ratio) of 4.91 and contained 13 (25%) of the amino acid positions, 8 of which overlap with the antigen binding sites defined by Brown *et al*. [41].

**Table 1:** Signatures of selection on the MHC class IIB loci.

Allele set	Region	# of aa sites	d_N_	d_S_	d_N_/d_S_	P
All alleles n = 28	ABS	15	0.44 ± 0.08	0.06 ± 0.03	**7.60**	**< 0.001**
	Non-ABS	42	0.13 ± 0.03	0.13 ± 0.04	1.00	0.998
	All	57	0.20 ± 0.03	0.11 ± 0.03	**1.78**	**0.018**

Alleles with deletion n = 8	ABS	15	0.19 ± 0.05	0.00 ± 0.00	**na**	**0.002**
	Non-ABS	42	0.09 ± 0.02	0.09 ± 0.04	0.94	0.865
	All	57	0.11 ± 0.02	0.06 ± 0.03	1.71	0.117

Rates of non-synonymous (d_N_) versus synonymous (d_S_) substitutions for the amplified part of exon 2 as well as for residues of the antigen binding site only. Data is shown for all alleles and separately for the eight alleles with the deletion. ABS - Antigen Binding Site. aa - amino acid. Significant values are marked bold.

### Effects of MHC diversity on body condition and parasite load

**Figure 3:**
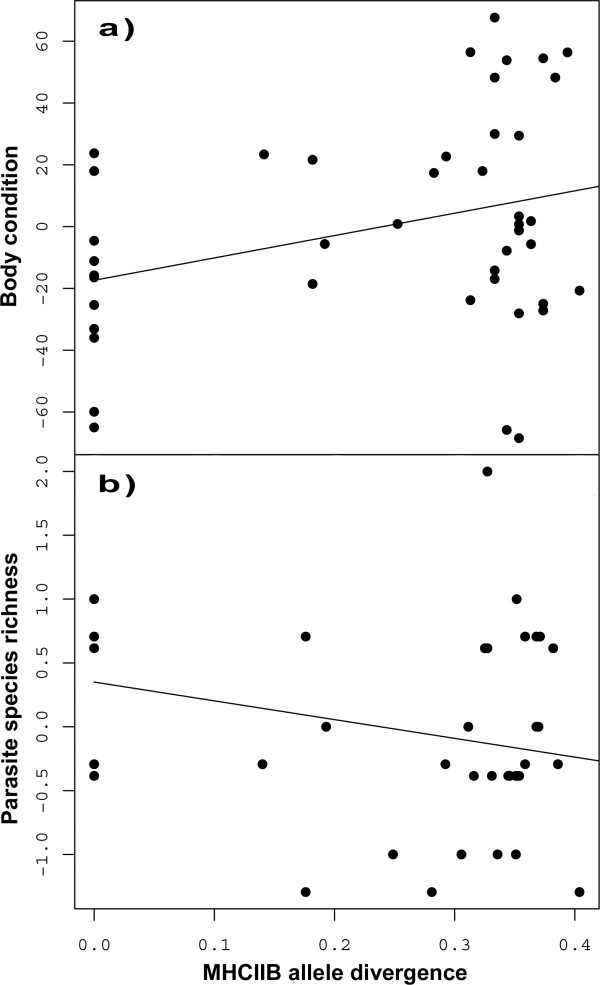
**Correlation of individual MHC IIB allele divergence (average amino acid genetic distance) with a) body condition (residuals of weight against length) and b) parasite species richness (residual number of helminth morphotypes)**.

Out of the 46 analysed individuals, 20 were categorized as adults, while the rest was classified as subadults. Only 23 of the 46 individuals were mature enough for sexing. Body condition was positively associated with individuals' allele divergence, i.e. average amino acid distance between alleles (ANCOVA, F_1,46 _= 5.09, p = 0.030, Fig. 3a), whereas study site, cohort or age class did not have any effect on body condition. ABS divergence, i.e. allele divergence at antigen binding sites only, showed the same effect (ANCOVA, F_1,46 _= 4.6, p = 0.039), while allele number showed only a slight positive trend in this respect (ANCOVA, F_1,46 _= 3.35, p = 0.075). However, the strongest effect originated potentially from the difference between individuals with a single allele and those with more than one (t-test, t = 3.6, p = 0.001). Sex did not influence body condition in the subsample (t-test, t = 1.4, p = 0.22).

**Figure 4:**
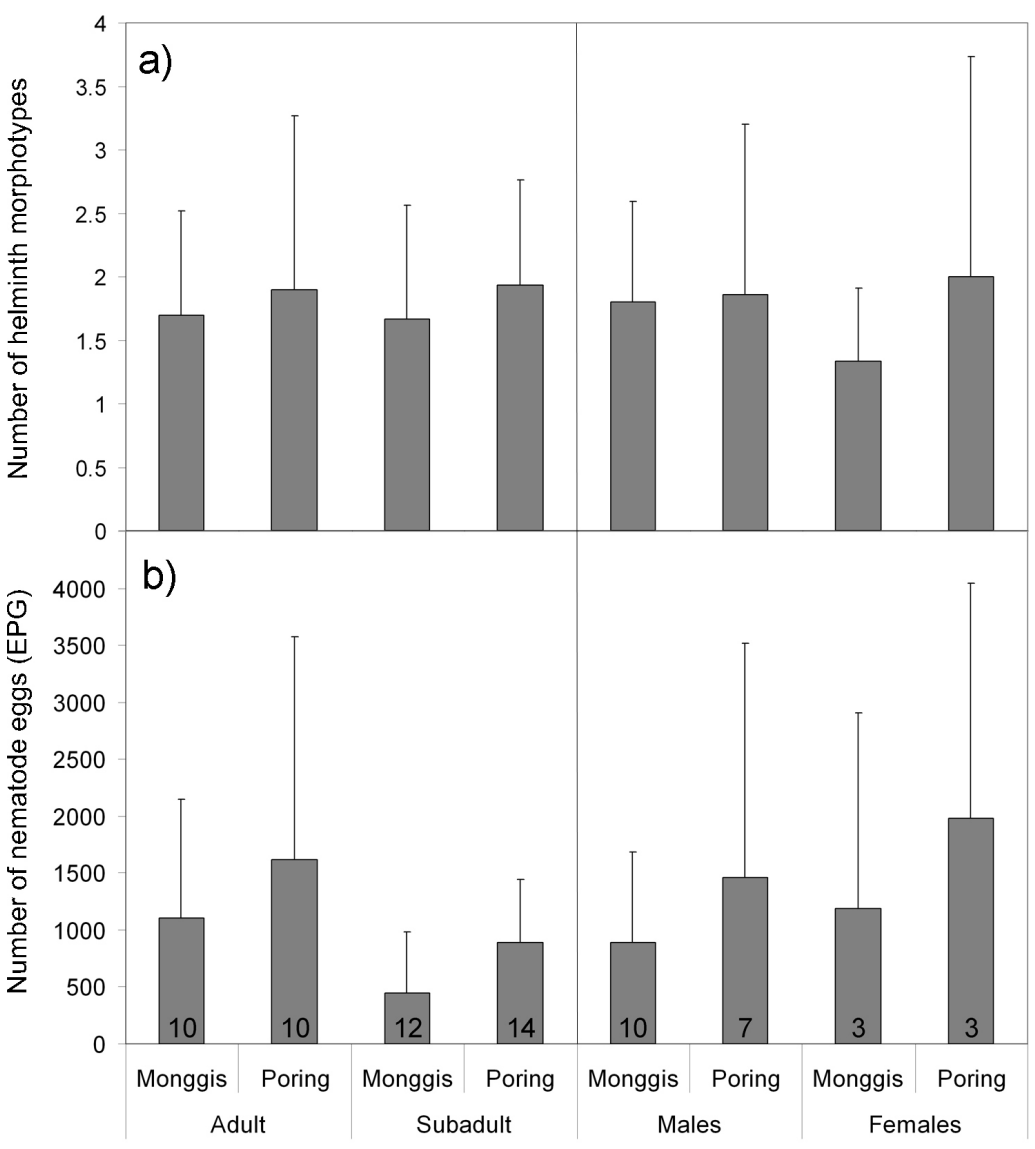
**Parasite load separated for sex and age in each sampling site**. For each sampling site (Monggis, Poring) the a) mean number of helminth morphotypes (parasite species richness) and b) infection intensity (nematode eggs per gram faeces, EPG) are shown. Error bars indicate standard deviation. Black numbers indicate sample size for each group (total N = 46). Note that sex could not be determined in some cases due to immaturity of individuals.

In total 11 different helminth morphotypes were identified (8 nematodes and 3 cestodes) with zero to four morphotypes per individual. The number of helminth morphotypes per individual did not differ between study sites, age or sex (Mann-Whitney U, all p > 0.24; Fig. 4a). The overall prevalence of nematodes was high with 91.3%, whereas cestodes were found in only 23.9% of the screened individuals and trematodes could not be detected at all. Cestodes were significantly more prevalent in the unlogged site with 44% (Poring) than in the logged site with 4.8% (Monggis, Yates corrected X^2^_1,43 _= 8.79, *p *= 0.003), whereas neither nematode prevalence (Likelihood ratio X^2 ^_1,43 _= 1.36, p = 0.24) nor intensity (Mann-Whitney U, p = 0.15; Fig. 4b) differed between the locations. We pooled the individuals for further analysis of those parameters that did not show any differences between study sites.

We found a significant negative influence of allele divergence on the number of helminth morphotypes (parasite species richness) within an individual (GLM; X^2^_1,38 _= 2.42, p = 0.042; Fig. 3b). ABS divergence showed a similar trend (GLM; X^2^_1,38 _= 2.16, p = 0.056), while neither the number of alleles (GLM; X^2^_1,37 _= 1.50, p = 0.11) nor body condition (GLM; X^2^_1,34 _= 0.01, p = 0.97) had an effect on the number of helminth morphotypes. Again, a part of the association between allele divergence and parasite species richness was potentially due to the difference between individuals with one and those with more than one allele, since the latter showed a lower parasite species richness (Mann-Whitney U, p = 0.013). The models showed also that the number of helminth morphotypes differed significantly between the cohorts (GLM; X^2^_1,39 _= 6.18, p = 0.014). The residuals of all four models were well normally distributed (Shapiro-Wilk test; all p ≥ 0.40).

When testing, whether the intensity of nematode infection (nematode EPG) was influenced by the presence of individual MHC-alleles, the parameters body condition, allele divergence and allele number were removed from the respective models according to AIC and therefore had no significant influence. In contrast, intensity of nematode infection differed between age classes, being higher in adult individuals, and was higher in Poring (negative binomial GLM; both p < 0.05). For statistical reasons we only tested the effect of those alleles that occurred at least in six individuals (6 alleles). Individuals with the second most common allele Lesa-MHCIIB*13 tended to show a decreased intensity of nematode infection (negative binomial GLM, X^2^_1,41 _= 5.076, p = 0.024; not significant with Bonferroni corrected p = 0.008 for multiple testing). The intensity of nematode infection was independent of the sex (Mann-Whitney U, p = 0.46).

## Discussion

In this study, the variability of the MHC class IIB genes in the Long-tailed giant rat (*Leopoldamys sabanus*) was investigated and its effect on the parasite infection analysed. The aim was to further unravel the evolutionary mechanisms that lead to the observed high polymorphism in MHC genes by investigating a natural population in the diverse and dynamic environment of a tropical rainforest. As expected from studies on other small mammals, the MHC of the Long-tailed giant rat was highly polymorphic. This was shown in the diverse allele pool of the two subpopulations (28 distinct alleles in 46 individuals) as well as in the high percentage (70%) of polymorphic amino acid sites within the exon 2, the domain that is responsible for antigen binding [41].

The inconsistent BLAST results might indicate that MHC IIB locus differentiation is less consolidated than sometimes assumed. Mammals are the only order where the differentiation between DRB and DQB loci is actually being made, while in other vertebrate orders the different MHCIIB loci are usually indistinguishable. Most studies use the NCBI BLAST search for identification of locus origin and this might be sometimes misleading, as locus identification is commonly referred from distantly related taxa if no species specific sequence data is available. The so identified sequences then function again for later studies as reference and artificially reinforce locus determination. BLASTing for instance a published DRB1 allele of *Canis familiaris *[GenBank:AF016910] to the dog genome [44] brings as first hit the DLA DQB1 locus. Additionally there is support for the occurrence of interlocus exchange of sequence motifs [45,46], which could also lead to misleading BLAST results.

The range of one to four alleles per individual suggests that the number of MHCIIB loci differs between haplotypes, albeit it is theoretically possible that the same allele occurs on several loci. The variation in MHCIIB loci number has already been resolved for several vertebrate species, including mammals [47-51] and seems to occur in the Long-tailed giant rat as well. Another explanation would be the presence of null alleles, meaning that we did not amplify all present alleles. However, the used primers are rather conservative, have been established before in several mammalian species and produced more than two alleles in some of them, indicating duplicated MHCIIB loci as well [22,52-56]. The puzzling deletion in some of the alleles is not species specific and can be found in some (but not all) DQB alleles of the Brown rat [57] and the House mouse (e.g. [GenBank:AY740472]). However this sequence characteristic alone is not enough to refer locus origin, as it is not deterministic for one of the loci. The Neighbour-joining tree does not solve the question of locus origin either, as none of the Lesa allele groups gets significant bootstrap support. These results suggest that the divergence between the two detected MHC class II loci is either hindered by frequent inter-locus recombination or by the fact that both loci evolve under the same selective forces. Under these circumstances the apparent lack of traces of inter- and intra-locus recombination events therefore suggests that both loci code for molecules that provide the same function and are shaped by the same selective forces. As done in most studies that analyse MHC diversity in natural populations, we amplified only the exon 2 of MHC IIB genes, since this codes for the antigen binding region of the MHC molecule. Despite high functional and structural similarity of the molecules and presumable paralogous origin, the different MHC IIB loci might have diverged more strongly in other exons. Restricting the comparison to the exon 2 sequence only might therefore contribute to the dissatisfying locus resolution. However, a recent meta-analysis on MHC in several rodent taxa, some of them with duplicated loci, confirmed that the overall combined number of alleles from several class II loci is shaped by local parasite species richness [58].

The observed high ratio of non-synonymous to synonymous substitutions is a strong sign for balancing selection and strengthens the assumption that both loci are functional and currently involved in antigen presentation, which is not always the case as seen in a recent study on MHC genes in bank voles [56]. This is supported by CODEML, which showed that the positive selection model M8 explained the obtained sequence data better than the model for neutrality M7. Overall, our results do not show any sign for non-functionality or pseudogenes and indicate that the analysed genes in this tropical rodent behave like classical MHC genes. We consequently assume that they are actively involved in antigen presentation, providing resistance against pathogens and confer a crucial adaptive trait for the Long-tailed giant rat. Therefore we analysed possible adaptive effects of the individual MHC-constitution on overall body condition as well as helminth parasite infestation to shed more light onto the selection mechanisms that lead to such high polymorphism.

Interestingly, the allele divergence (average genetic distance of alleles on the amino acid level within an individual) was correlated with a lower individual number of parasite morphotypes and a better overall body condition. This is in line with the *'divergent allele advantage*' hypothesis [34], which assumes that individuals with more divergent alleles have an advantage, because their immune system can counteract against more different pathogens and therefore leads to '*overdominant selection*' [29,30]. Other studies have found an unexpected high divergence of MHC alleles in natural populations [59] and also signs for '*divergent allele advantage*' in the context of mate choice [60-63]. Consuegra & Garcia de Leaniz [61] also found a positive effect of MHC dissimilarity on the prevalence of a single parasite. However, the current data set does not provide enough statistical power to disentangle the effects of allele divergence and pure heterozygote advantage.

The question remains whether the sequence divergence between alleles is a better functional predictor for immunocompetence than the plain number of alleles. It makes intuitively sense to give more importance to allele pairs that differ by 30 amino acids than to those that differ by only one amino acid. Studies that experimentally support the idea of overdominance in allele numbers [e.g. [26], up to an optimum in [27]], may in fact show indirectly the effect of allele divergence, which is supported by our observation that the genetic divergence within an individual correlates strongly with the number of alleles.

The genetic divergence increases strongest with the step from one allele to two alleles. Every additional allele thereafter only adds a fraction of its antigen binding potential, since alleles are known to overlap in their antigen repertoire [64] and each new allele only adds the fraction of antigens that are not yet bound by the already present alleles. The observed correlation between MHC allele divergence and parasite species richness might to a significant part be due to the observed difference between individuals with one allele and those with more alleles. This is, however, not the only reason for the correlation, since we would then also expect a stronger correlation of parasite species richness and mere allele number, which was not detected.

Our finding of a potential association between intensity of nematode infection and a certain allele, which looses significance after Bonferroni-correction, would support the *'negative frequency-dependent selection*' hypothesis, which has already been shown in several other host parasite systems [16,18,20,54,65,66]. The fact that the potentially 'resistant' allele Lesa-MHCIIB*13 is the second most common in our population, although a rather rare allele would be expected by this hypothesis, could be due to the sampling time point. The cycling pattern of frequencies of both alleles and parasites due to host-parasite coevolution can lead to a spread of an advantageous and formerly rare allele until the parasite evolves to avoid it [67]. The *'negative frequency-dependent selection*' hypothesis could also explain the maintenance of the observed high number of alleles that were found in one or two individuals only. It expects a potential advantage for individuals carrying rare alleles, which enable them to better resist new pathogen threats and therefore keeps those rare alleles in the gene pool instead of purging them.

## Conclusion

The negative correlation of mean genetic distance between MHC IIB alleles and the number of parasite morphotypes could be interpreted in support of the '*divergent allele advantage*', which has to our knowledge not yet been fully proven by empirical studies. However, the main effect originates likely from the difference between individuals with one and those with more alleles. In addition we found a substantial number of rare alleles and hints for an interaction between a specific allele and nematode infection intensity. We therefore conclude that both hypotheses, the heterozygosity advantage (and potentially the '*divergent allele advantage*' as a special case thereof) as well as the '*negative frequency-dependent selection*', work non-exclusively on the maintenance of a high variability in the MHC allele pool, as it has already been proposed in theory by Apanius *et al*. [3]. More work has to be done on the molecular level to discover and unravel the interrelations of individual alleles and individual parasite genotypes to finally resolve the mystery of balancing selection on the MHC loci. A special focus should be put on the importance of individual allele divergence, e.g. by looking at a broader parasite range.

## Methods

### Study area and animal collection

The samples were collected in two lowland rainforest sites near Mount Kinabalu in the federal state of Sabah in Malaysia, North-western Borneo. While one site ('Poring') was located in an old-growth forest area within the Kinabalu National Park (6°22'N, 116°42'E), the other site ('Monggis') was located in a distance of ca. 21 km beeline in an adjacent logged forest area near the village Monggis (6°13'N, 116°45'E), which has been logged about 20 years ago. Both forest areas comprised more than 1000 hectares. Animals were captured in four trapping sessions (cohorts) between 2002 and 2004 with wire-mesh live traps placed along transects of 380 meter length and additional traps as described in Wells *et al*. [68].

Traps were baited with ripe banana and checked every morning. Captured individuals were weighed and first-capture individuals were anaesthetised with diethyl ether for further handling. We injected a subcutaneous transponder (AEG Identification Systems) for permanent identification. Then animals were aged, sexed and morphological measurements (weight, body length) were taken. We sampled ear tissue with a biopsy ear punch and stored it immediately in 95% ethanol. Faeces were collected from the bottom of the trap and stored in 2-3% formalin. Animals were handled and immediately released at the point of capture. The animal handling and sampling protocol followed guidelines of the American society of Mammalogists [69] and was approved by the Economic Planning Unit Malaysia and the Sabah Parks authority.

### Parasite screening

We focused on helminth parasite species because their prevalence and the intensity of infestation can be assessed non-invasively from faecal samples. We counted all eggs from nematodes, cestodes and trematodes (Plathelminthes) from faeces with a modified flotation and McMaster method [19,68,70]. This non-invasive technique has been shown to be accurate for quantification of helminth eggs (e.g. [71-73]). Samples of approximately 600 mg faeces were dissolved in 9 ml potassium iodide solution (specific gravity 1.5 g/ml), sieved to remove large debris and screened for helminth eggs by counting the content of two chambers of a McMaster slide. We calculated infection intensity as eggs per gram faeces (EPG). All eggs were photographed and measured (Zeiss, AxioCam and AxioVision software; 10-40× amplification). Images were assigned to operational taxonomic units (orders for nematodes, cestodes) based on features of egg shell and plasma, and further distinguished into morphotypes by size classes and shell thickness for strongyle nematodes [68,74]. The overall number of helminth morphotypes within each rat was then used as a measure of parasite species richness.

### Molecular techniques

DNA was extracted according to the mouse tail protocol using the DNeasy Tissue Kit (Qiagen, Hilden). We aimed at amplifying the highly variable exon 2 of the MHC class IIB genes. As there was no sequence data available for the Long-tailed giant rat, we used the well established primers JS1: 5'-GTGTCATTTCTACAACGGGACG-3' [53] and GH50: 5'-CTCCCCAACCCCGTAGTTGTGTCTGCA-3' [52], which have been established for DRB loci in small mammals. When BLASTed against the NCBI nr database, the first 1000 hits for both primers deliver only MHC II DRB genes from different species (searching for distant homologies and adjusting for short sequence queries). These primers lead to a fragment of 221 bp length, which spans most of the exon 2, including the most polymorphic part of the MHC IIB gene. Reactions of 20 μl contained 2-4 μl template DNA, 2 μl 10× incubation buffer incl. MgCl_2_, 0.175 mM of each dNTP, 1 unit taq polymerase (all QBioGene, Irvine) and 0.375 mM of each primer (MWG Biotech, Ebersberg). PCR was performed on a TGradient Thermocycler (Biometra, Göttingen) with an initial denaturing step of 96°C for 2 min, 34 cycles of 96°C for 30 s, 54.7°C for 60 s and 72°C for 60 s and a final elongation step of 72°C for 10 min. We checked the PCR product for quality and size on a 1.5% agarose gel and then purified using the QIAquick Gel Extraction Kit (Qiagen, Hilden) according to the manufacturers protocol, but eluting in dH_2_O. An additional ethanol precipitation was done to further purify the product.

Alleles were separated using Single Stranded Conformation Polymorphism (SSCP [75]). It is a sensitive method to distinguish even minimal allele differences [76,77], which has been widely used in human genetics and became popular in population genetics and evolutionary ecology as well [19,27,62,78-80].

Products were denatured at 95° for 5 min and immediately transferred to ice for snap-cooling to produce single-strands and hinder reannealing. The ssDNA was then mixed with loading dye and loaded on a non-denaturing polyacrylamide gel (CleanGel HP-15 from ETC, Kirchentellinsfurth) according to the manufacturer's protocol and with the following adaptations: 12°C running temperature, pre-run for 20 min at 200 V, 20 mA, 10 W and main run for 4.5 h at 450 V, 30 mA, 20 W.

Gels were silver stained, scanned and processed with Photoshop 7.0 (Adobe Systems Inc, Beaverton) to align individual's band patterns. We counted bands with the same mobility as same alleles and each of these alleles was sequenced at least once from both directions, if possible twice from different individuals, to confirm this assumption. Appropriate bands were cut from the polyacrylamide gel, eluted in TBE buffer, amplified with the same protocol as above but only 30 cycles and using 1-2 μl of eluate. The PCR product was gel purified, ethanol-precipitated as above and then sequenced using the chain termination method [81] and either the JS1 forward or the GH50 reverse primer.

Sequences were aligned manually and obvious PCR misincorporations (unreproducible SNPs) were omitted. Alleles were only counted when they could be supported by two independent sequences from one individual or, if possible, from two different individuals. Primer sequences were excluded from analysis.

### Genetic analysis and statistical treatment

Sequence alignment was done manually with the sequence analysis software BioEdit [82]. We used GENECONV [83], a powerful tool for the detection of sequence recombination [84], to test for traces of gene conversion events within and between loci. A phylogenetic tree was built with MEGA 4 [85] based on neighbour-joining algorithm and nucleotide distance. The distance was estimated in MEGA 4 with a maximum composite likelihood method [86] based on a model by Tamura & Nei [87]. This model corrects for multiple hits, takes into account the differences in substitution rates between nucleotides and the inequality of nucleotide frequencies and also distinguishes between transitional and transversional substitution rates.

We used the software MEGA 4 to calculate d_N_/d_S _ratios and the therein implemented Z-test to test for signs of selection. This was done for the full sequence and separately for those sites only that are directly involved in antigen binding (ABS) [41]. To confirm this selection estimate we also performed a model test with CODEML. This tool is included in the PAML4 package [88] and calculates maximum likelihood estimates for the fit of models with different assumptions of selection patterns to the sequence data. We tested the models M7 (neutral) against M8 (positive selection). The models are described in detail in Yang *et al*. [88] and can be compared using the likelihood ratio test by calculating the likelihood difference 2Δl = 2(l_1_-l_0_) and compare it to a X^2^-distribution with the degree of freedom equal to the difference in the number of estimated parameters [89].

As a measure of MHC diversity besides the pure number of allelic variants, we determined allele divergence, i.e. the average genetic distance between all MHC IIB alleles of an individual. This distance was calculated in MEGA 4 as the sum of all pairwise amino acid p-distances between all alleles of an individual, divided by the number of possible pairings (2 alleles = 1, 3 alleles = 3, 4 alleles = 6). The p-distance was calculated according to the model of Tamura & Nei [87]. The genetic distance for individuals with only one allele is zero. The same estimate for allele divergence was also calculated taking only the antigen binding sites into account (ABS divergence). Correlation between allele number and average distance was tested with Spearman rank correlation because of non-normality of the data.

Body condition as a measure of physical fitness is difficult to estimate in a living animal [90,91]. Therefore we used an approximation index, taking the residuals from the regression of body mass against body length [90]. To test effects of study site, cohort (time of sampling), age and MHC diversity on body condition, we used linear models (ANCOVA) and the step function in the statistical software R, version 2.6 [92], which selects the most appropriate model based on Akaike's information criterion (AIC). Three different estimates for MHC diversity were used independently because of co-linearity: allele number, allele divergence and ABS divergence. Normality of response variable and independence of included factors was verified. Sex could only be determined in a subsample of the individuals and was therefore left out of the models. However, we tested the effect of sex on body condition and parasite load (species richness and infection intensity) within this subsample using t-test or Mann-Whitney U tests. For analysis of infection parameters we included only parasitized individuals (n = 43). Since it is unlikely to find individuals without a single parasite egg (lowest EPG count in parasitized individuals = 50), we feared that the egg count was confounded in those three individuals and might deteriorate the results.

Parasite species richness within each individual, expressed as number of different helminth morphotypes, was not normally distributed (Shapiro-Wilk test, p < 0.001). Therefore we used a generalized linear model (GLM, quasipoisson distribution, log link). We analysed the effect of study site, cohort, age and MHC diversity on parasite species richness. Again we tested allele number, allele divergence and ABS divergence independently. The model was optimised manually, because the step function is not available for models with quasipoisson distribution. The effect of body condition was tested in a separate model without MHC diversity because of co-linearity. The validity of the chosen models was evaluated by testing the distribution of the residuals.

To analyse the infection intensity in more detail we focussed on nematodes, because of their high prevalence. Effects of study site, cohort, age and MHC diversity on nematode infection intensity (EPG of all nematode morphotypes pooled) were tested with a negative binomial GLM with log link (glm.nb function in the MASS package, [93]), because of its distribution. Again the step function was applied to find the best fitting model. The effect of allele number, allele divergence and body condition on infection intensity was tested in independent models because of co-linearity. To analyse the effect of individual alleles, they were included singly in the GLM with the other factors. A Bonferroni correction had to be applied for testing multiple alleles. All tests were performed two-tailed using R 2.6 [92] or SPSS 13 (SPSS Inc., Chicago) and test assumptions were verified. All tests are also assigned in the results section.

## Authors' contributions

SS supervised the study. KW, MP and SS designed the field- and parasitological work. KW and TLL collected the samples. KW carried out the parasitological work. TLL and SS designed the lab work. TLL carried out the molecular work, performed the statistical analysis and drafted the manuscript. SS, KW and MP helped to draft the manuscript. All authors read and approved the final manuscript.
